# The Impact of Adenotonsillectomy on the Quality of Life of Children with Obstructive Sleep Apnea

**DOI:** 10.1055/s-0044-1786832

**Published:** 2024-10-25

**Authors:** Geethi Krishna Sukumaran, Asha Chellappan Sunanda, Shajul George

**Affiliations:** 1 Department of ENT, Government Medical College, Kottayam, Kerala, India

**Keywords:** adenoidectomy, tonsillectomy, sleep apnea, obstructive, quality of life

## Abstract

**Introduction** In children, obstructive sleep apnea (OSA) is a sleep-related breathing disorder that is caused by adenotonsillar hypertrophy and is characterized by upper airway obstruction disturbing sleep.

**Objective** We conducted this study to evaluate health-related quality of life (QoL) in children with OSA before and after adenotonsillectomy.

**Methods** A descriptive, observational study was conducted among 43 children in the 4-to-12 years old age group who had symptoms of OSA due to adenotonsillar hypertrophy and who underwent adenotonsillectomy at a tertiary care center during the period from February 2020 to February 2021. The QoL was assessed using the OSA-18 questionnaire preoperatively and at 2 and 6 months postoperatively.

**Results** Among the study population, males (72)%) were more affected with OSA, with a male-to-female ratio of 2.6:1. Based on the OSA-18 questionnaire assessment, the most severe and frequently observed symptoms were in the domains of sleep disturbance and physical symptoms, in which the mean score was 77 preoperatively. After adenotonsillectomy, the mean OSA-18 score decreased to 28.605 and 22.465 at 2 and 6 months, respectively. At 2 months postsurgery, more significant improvement was noticed in sleep disturbances, physical symptoms, and parent's concern while at 6 months postsurgery, all domains showed equal improvement. Therefore, following adenotonsillectomy, the QoL improved significantly.

**Conclusion** Obstructive sleep apnea can adversely affect sleep quality as well as neurocognitive and cardiovascular functions. Adenotonsillectomy resulted in significant improvement in the QoL.

## Introduction

Sleep disorders in children include primary snoring, upper airway resistance syndrome, and obstructive sleep apnea (OSA). Obstructive sleep apnea is characterized by episodes of upper airway collapse or obstruction during sleep leading to apnea or hypopnea that fragments sleep. The prevalence of OSA is around 1 to 3%. Diagnosis can be established by thorough history taking, examination, and sleep studies. The gold standard method to diagnose and assess the severity of OSA is polysomnography. The most common cause for OSA in children is adenotonsillar hypertrophy. The condition is usually manifested in the 2-to-6 years old age group as it is the period during which the adenoid and tonsils reach their maximum size. So, adenotonsillectomy can be considered as the first-line treatment of choice for pediatric OSA. The consequences of long-term sleep apnea include cardiovascular dysfunction as well as neurocognitive and behavioral problems.

The quality of life (QoL) in children with OSA can be assessed with the OSA-18 questionnaire, which is widely used nowadays. The questionnaire contains 5 domains and a total of 18 items. The domains include physical symptoms, sleep disturbances, emotional distress, daytime symptoms, and parents' concerns. The domains of physical symptoms, sleep disturbances, and parents' concerns contain four items while the other two domains contain three items in each. To grade the severity of the problem addressed in each item, a score of 1 (none of the time) to 7 (all the time) is used. The study was conducted to assess the impact of adenotonsillectomy on the QoL in children with OSA.

## Methods

This descriptive observational study was conducted among 43 children with symptoms of OSA due to adenotonsillar hypertrophy and who underwent adenotonsillectomy at a tertiary care center during the period from February 2020 to February 2021. They were in the age group of 4 to 12 years old. The informed written consent was obtained from the parents/guardians of the children included in the study once institutional ethics committee clearance was received. Children younger than 4 years old, those with nasal obstruction due to other causes (such as deviated nasal septum), congenital nasal deformities, allergic rhinitis, and children with cleft palate and submucous cleft were excluded.

Using a pretested structured proforma, the relevant information was obtained. Detailed history and ear, nose, and throat (ENT) examination were performed. The OSA-18 questionnaire was administered to those included in the study to assess the physical symptoms, sleep disturbances, emotional distress, daytime problems, and parents' concerns a day before surgery and at 2 and 6 months after the surgery. The total score was 126; a score of 0 to 60 indicated small impact on the QoL, a 60-to-80 score denoted moderate impact, and a score greater than 80 indicated severe impact on the QoL. The relevant data were collected and formatted into tables and charts. Data were coded and entered on a Microsoft Excel (Microsoft Corp., Redmond, WA, USA) worksheet and analyzed using the SPSS for Windows, version 16.0 (SPSS Inc., Chicago, IL, USA). For all quantitative variables the mean and standard deviations were computed. Using a repeated-measures analysis of variance (ANOVA), preoperative and postoperative OSA-18 scores were compared. A *p* - value < 0.05 was taken as the level of significance.

## Results

Among the total study population (43), the majority were in the age group of 7 to 12 years, that is 29 children (67.44)%), whereas 14 (32.56)%) children were in the 4 to 6 years age group. Thirty-one (72.09%) children were male and 12 (27.91%) were female. Among the total population, 21 had symptom for 4 to 6 years, 15 (34.9%) had symptoms for ∼ 1 to 3 years, and 7 (16.3%) had symptoms for more than 6 years. Thirty-nine children used nasal sprays for more than 6 months and 4 of them used nasal spray only for less than 6 months. A total of 33 (76.7%) had adenoid facies, and 10 children (23.3%) had no features of adenoid facies. Out of all participants, ∼ 25 (58.1%) had grade-3 tonsillar enlargement, 11 (25.6%) had grade-4, and 7 (16.3%) had grade-2 tonsillar enlargement. A repeated-measures ANOVA determined that the mean OSA scores differed significantly across the 3 time points (F = 528.519, *p* = 0.001). A posthoc pairwise comparison using the Bonferroni correction showed a decrease in the OSA score between the initial assessment and the follow-up ones (77,28.605 and 22,465, respectively); this is statistically significant ( *p* = < 0.001). Therefore, we can conclude that the results of the ANOVA indicate a significant OSA-18 score reduction following the surgical procedure ( Table 1 ).

**Table 1:** Comparison of preoperative, 2-month, and 6-month postoperative OSA-18 scores

Repeated-measures ANOVA analysis				
Estimates				
	Mean	Std. error	95% confidence interval	
			Lower bound	Upper bound
OSA-18 scores preoperatively	77	1.73	73.49	80.50
OSA-18 scores 2 months postsurgery	28.605	0.90	26.77	30.43
OSA 18 scores 6 months postsurgery	22.465	0.45	21.54	23.38
Pairwise comparisons				
Factors		Mean difference	Std. error	*P* -value
OSA-18 scores preoperatively	OSA-18 scores 2 months postsurgery	48.395	1.555	< 0.0001
	OSA-18 scores 6 months postsurgery	54.535	1.688	< 0.0001

Abbreviation: ANOVA, analysis of variance.

Among the 5 domains, sleep, physical, and parent concern are the ones with the highest difference 16, 14.23, and 11.83, respectively, at 2 months. At 6 months, almost all domain showed marked improvement ( *p* < 0.001) ( Tables 2 , 3 , 4 , 5 , and 6 ).

**Table 2:** Comparison of the sleep disturbances domain preoperatively, and at 2 and 6 months postsurgery

Pairwise comparisons				
Sleep domain		Mean difference	Std. error	Significance
Preoperatively	At 2 months	16	0.427	< 0.001
	At 6 months	17.698	0.441	< 0.001

**Table 3:** Comparison of the physical symptoms domain preoperatively and at 2 postop and 6 months postsurgery

Pairwise comparisons				
Physical domain		Mean difference	Std. error	Significance
Preoperatively	At 2 months	14.233	0.514	< 0.001
	At 6 months	16.907	0.491	< 0.001

**Table 4:** Comparison of the emotional disturbances domain preoperatively and at 2 postop and 6 months postsurgery

Pairwise comparisons				
Emotional domain		Mean difference	Std. error	significance
Preoperatively	At 2 months	2.279	0.33	< 0.001
	At 6 months	2.814	0.374	< 0.001

**Table 5:** Comparison of the daytime symptoms domain preoperatively and at 2 postop and 6 months postsurgery

Pairwise comparisons				
Daytime symptoms domain		Mean difference	Std. error	Significance
Preoperatively	At 2 months	5.86	0.464	< 0.001
	At 6 months	5.86	0.464	< 0.001

**Table 6:** Comparison of the parent's concern domain preoperatively and at 2 postop and 6 months postsurgery

Pairwise comparisons				
Parent concern domain		Mean difference	Std. error	Significance
Preoperatively	At 2 months	11.837	0.605	< 0.001
	At 6 months	11.837	0.605	< 0.001

## Discussion

Obstructive sleep apnea can affect the neurocognitive behavior of children as well as contribute to daytime symptoms like hyperactivity and aggressive behavior. 1 It can also lead to metabolic and cardiovascular abnormalities significantly affecting QoL . 2 3 The OSA-18 questionnaire is a widely used tool to assess the QoL in children with OSA. 4 Based on literature reviews, adenotonsillectomy is the preferred modality of treatment for children with OSA due to adenotonsillar hypertrophy. 5 6 7 There is significant improvement in QoLin children following adenotonsillectomy when compared with those who had conservative management. A total of 67.4% of our study population were in the age group of 7 to 12 years, with an average age of 7.8 years. This is higher than that reported by Ahamed et al., who found an average age of 5.44 years, and a median age of 5 years. 8 According to our study, OSA was more prevalent in males (72%) than in females ((28)%), with a male-to-female ratio of 2.6:1. This was comparable to the results of other similar studies conducted by De Serres et al. and Mitchell et al. 9 10 11 Of the total study population, 25 (58.1%) had grade-3 tonsillar enlargement, 11 (25.6%) had grade-4, and 7 children (16.3%) had grade-2 tonsillar enlargement. The OSA 18 questionnaire was developed by Franco Jr et al. to assess the impact of OSA on the QoLof children, and it is used to assess the QoLat 3 different intervals (preoperatively, 2 months, and 6 months postoperatively). According to Franco Jr et al., patients who scored less than 60 had a small impact on their QoL, those who scored between 60 and 79 had moderate impact on QoL, and those who scored 80 points or above had a severe impact on their QoL. 12 The improvement in the QoL can be assessed by the decrease in the scores for each domain of the OSA-18 as well as by the changes in the total score, which is present in the current study and in previous studies. 13 The improvement in QoL in children with OSA after adenotonsillectomy was maintained for at least 6 months in our study, which is similar to the finding in the study by Mitchell et al. 14 The mean changes in the score at 2 and 6 months postoperatively compared with the preoperative score were significant based on statistical analysis ( *p* < 0.0001). The OSA-18 mean score before surgery was 77 and it decreased to 28.605 at 2 months and 22.465 at 6 months postoperative. This revealed that, preoperatively, OSA had moderate impact on the QoL in the majority of study population, and after the surgery there was only a small impact on the QoL. De Lima Junior et al., in their study of 48 children, found that before surgery, the mean OSA-18 score was 82.83 (SD = 12.57). Within 1 month after surgery, the mean OSA-18 score was 34.3 (SD = 9.95), which showed a marked reduction ( *p* < 0.001). According to their study, surgery improved the QoL significantly in children with OSA, which was similar to the results of our study. 15 Among the 5 domains, sleep, physical symptoma, and parent concern are the ones with the highest difference 16, 14.23, and 11.83, respectively at 2 months ( *p* < 0.001). That is, sleep disturbances, physical symptoms, and parent concern domains showed maximum improvement at 2 months after adenotonsillectomy when compared with emotional disturbances and daytime symptoms domain. Valerie et al., in their study, used the OSA-18 questionnaire to assess QoL. Postoperatively, the OSA-18 scores of all domains showed improvement, which was consistent with our study. Franco et al., in their study, also found that there was a significant improvement in QoL in patients who had undergone adenotonsillectomy for OSA, which was similar to our study. Long-term follow-up after adenotonsillectomy is difficult as most patients do not require further medical check-ups after improvement of the symptoms. This is applicable in children without comorbidities, since the surgical success rate in this group has been found to be high. Some studies describe recurrence of symptoms during the follow-up, especially in female children with normal weight and older than 6 years old, which was attributed to an incomplete therapeutic response and due to a change in the parental perception of symptoms. In our study, none of the patients presented worsening of the OSA-18 score after surgery up to 6 months. The Sleep Disturbance Scale for Children (SDSC) and Conners' Parent Rating Scale Revisited are good instruments to assess the presence of neurocognitive, neurobehavioral, or sleep disturbances associated with OSA. This in combination with clinical history, accurate physical examinations, and instrumental tests are necessary to diagnose or rule out OSA in children. Furthermore, there was a significant improvement of the neurocognitive, neurobehavioral, and sleep disturbances after surgery, thus demonstrating the efficacy of adenotonsillectomy in the treatment of the neurocognitive disorders associated with OSA in children. 16

## Conclusion

Pediatric OSA is predominantly seen in the 4-to-12 years age group, with male children being affected more frequently. The impact of OSA on the QoL of children can be assessed by disease specific questionnaires, such as the OSA-18. The OSA-18 questionnaire consists of 5 domains of sleep disturbances, physical symptoms, emotional distress, daytime problems, and parents' concerns. Obstructive sleep apnea can adversely affect sleep quality, neurocognitive functions, and cardiovascular functions. Adenotonsillar enlargement is one of the leading causes for OSA in children. Removal of the adenoid and tonsils has resulted in significant reduction in OSA-18 score from a preoperative score of 77 to 28.605 at 2 months, and, at 6 months postop, the average score was 22.465. Preoperatively, OSA had moderate impact on the QoL in most of the study population, and, after surgery up to 6 months, OSA had only mild impact on the QoL. Sleep disturbances, physical symptoms, and parents concern domains showed maximum improvement at 2 months after the surgery, and, at 6 months, almost all domains showed similar improvement. Thus, we can conclude that adenotonsillectomy is one of the best interventions to improve the QoL in children with OSA due to adenotonsillar enlargement.
